# Treatment Strategies in Managing Negative Symptoms in Schizophrenia

**DOI:** 10.1192/j.eurpsy.2025.2271

**Published:** 2025-08-26

**Authors:** S. Y. Teoh

**Affiliations:** Department of Psychological Medicine, National University of Singapore, Singapore, Singapore

## Abstract

**Introduction:**

Negative symptoms pose a significant challenge for the treatment and management of schizophrenia. They refer to the loss or diminishment of normal emotional and behavioural functions, which profoundly impact one’s quality of life and socio-occupational outcomes. They are often persistent and difficult to treat.

**Objectives:**

To explore and assess different treatment strategies for addressing negative symptoms of schizophrenia, including pharmacological, psychosocial, and non-invasive neurostimulation interventions. The goal is to provide an overview of current evidence and recommendations for enhancing the quality of life and functional outcomes in individuals with schizophrenia.

**Methods:**

We conducted a review of the extant literature to determine treatment strategies for negative symptoms in schizophrenia. We incorporated findings from randomised controlled studies, meta-analyses and systematic reviews.

**Results:**

We have identified several treatment strategies for negative symptoms in schizophrenia. The literature indicates that second generation antipsychotics such as Cariprazine and Amisulpride are associated with better functional outcomes with lower cognitive impairment. Adding on an anti-depressant, particularly to first-generation antipsychotics, has demonstrated positive effects. Psychosocial interventions including Cognitive Remediation (CR), Social Skills Training (SST) and exercise programs also alleviate negative symptoms. Additionally, non-invasive neurostimulation intervention such as rTMS applied to the left dorsolateral prefrontal cortex (DLPFC) has shown encouraging results in reducing negative symptoms.

**Conclusions:**

The findings highlight the important of comprehensive and holistic treatment approach integrating both pharmacotherapy and non-pharmacotherapy strategies to address the heterogeneity of negative symptoms. There is a need for further research into personalised treatments that address individual symptom profiles.

**Disclosure of Interest:**

None Declared

